# Identification of a 2′-*O*-Methyluridine Nucleoside Hydrolase Using the Metagenomic Libraries

**DOI:** 10.3390/molecules23112904

**Published:** 2018-11-07

**Authors:** Agota Aučynaitė, Rasa Rutkienė, Daiva Tauraitė, Rolandas Meškys, Jaunius Urbonavičius

**Affiliations:** 1 Department of Molecular Microbiology and Biotechnology, Institute of Biochemistry, Life Sciences Center, Vilnius University, LT-10257 Vilnius, Lithuania; agota.aucynaite@bchi.vu.lt (A.A.); rasa.rutkiene@bchi.vu.lt (R.R.); daiva.tauraite@bchi.vu.lt (D.T.); rolandas.meskys@bchi.vu.lt (R.M.); 2 Department of Chemistry and Bioengineering, Vilnius Gediminas Technical University, LT-10223 Vilnius, Lithuania

**Keywords:** metagenomics, nucleoside hydrolase, 2′-*O*-methyluridine, 2′-*O*-alkyl nucleosides, 5-fluorouracil

## Abstract

Ribose methylation is among the most ubiquitous modifications found in RNA. 2′-*O*-methyluridine is found in rRNA, snRNA, snoRNA and tRNA of *Archaea*, *Bacteria*, and *Eukaryota*. Moreover, 2′-*O*-methylribonucleosides are promising starting materials for the production of nucleic acid-based drugs. Despite the countless possibilities of practical use for the metabolic enzymes associated with methylated nucleosides, there are very few reports regarding the metabolic fate and enzymes involved in the metabolism of 2′-*O*-alkyl nucleosides. The presented work focuses on the cellular degradation of 2′-*O*-methyluridine. A novel enzyme was found using a screening strategy that employs *Escherichia coli* uracil auxotroph and the metagenomic libraries. A 2′-*O*-methyluridine hydrolase (RK9NH) has been identified together with an aldolase (RK9DPA)—forming a part of a probable gene cluster that is involved in the degradation of 2′-*O*-methylated nucleosides. The RK9NH is functional in *E. coli* uracil auxotroph and in vitro. The RK9NH nucleoside hydrolase could be engineered to enzymatically produce 2′-*O*-methylated nucleosides that are of great demand as raw materials for production of nucleic acid-based drugs. Moreover, RK9NH nucleoside hydrolase converts 5-fluorouridine, 5-fluoro-2′-deoxyuridine and 5-fluoro-2′-*O*-methyluridine into 5-fluorouracil, which suggests it could be employed in cancer therapy.

## 1. Introduction

Natural modified nucleotides are present in various kinds of nucleic acids and are most diverse in tRNA [1]. Functions of the nucleotide modifications span from providing structural stability and increasing resistance to physiological degradation of nucleic acids to transcriptional regulation and even implications in the regulatory pathways of the cell [2,3,4,5,6,7,8,9,10,11]. The vast variety of these modifications makes it difficult to elucidate the functions and biosynthesis of every single one of them. Nonetheless, the biosynthetic pathways are now rather well understood [12,13,14,15,16,17,18,19], albeit not all of them are deciphered completely. On the other hand, the studies of modified nucleic acid degradation are limited. “In Nature, everything that is made is unmade. Thus, for every pathway for the biosynthesis of a given metabolite there are complementary transformations for degradation and recycling of these building blocks back into living organisms” [20]. The biodegradation of modified nucleotides is understood mainly to the point of formation of nucleosides, even their subsequent conversion into heterocyclic bases is seldom described, with an exception of pseudouridine [21]. Despite of only currently emerging understanding of the full range of physiological functions of modified nucleotides, they are already exploited in medicine. The naturally occurring nucleosides are involved in numerous biological processes and serve as essential building blocks for both DNA and RNA, which provides a unique starting point for nucleotide analogue drug design. Hence, the nucleoside analogues are already used in the treatment of diseases like viral infections [22] and cancer [23]. Namely, the 2′-*O*-methyl RNA is a desirable modification to DNA aptamers [24] that are intended for therapeutics, diagnostics and have many other implications. The ribose 2′-*O*-methylation increases the hydrophobicity of nucleotides and protects them against the action of nucleases [25,26], which is a desired trait, especially for, e.g., DNA aptamers [24]. Despite the countless possibilities of practical use for the metabolic enzymes associated with methylated nucleosides, there are very few reports regarding enzymes involved in the metabolism of 2′-*O*-alkyl nucleosides [27,28,29]. The presented work focuses on the cellular degradation of 2′-*O*-methyluridine that is found naturally in rRNA, snRNA, snoRNA and tRNA of *Archaea*, *Eubacteria*, and *Eukaryota* [1].

## 2. Results

### 2.1. Discovery of RK9 Nucleoside Hydrolase and Deoxyribose-Phosphate Aldolase

**Figure 1:**
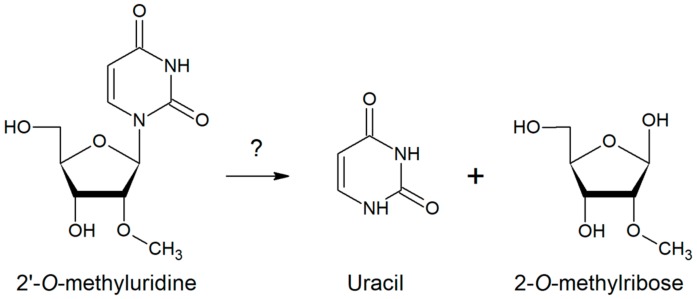
A schematic representation of the chemical reaction catalyzed by the unknown nucleoside hydrolase (represented with a question mark).

In order to select the genes, encoding the enzymes that participate in the metabolism of 2′-*O*-methyluridine, a screening system described previously [30] was used. We hypothesized that an *E. coli* strain lacking the UMP de novo synthesis pathway (the uracil auxotroph DH10BΔ*pyr*) would grow in the synthetic minimal media, if 2′-*O*-methyluridine (a sole source of uracil) would be converted into uracil (Figure 1) and subsequently used in the UMP salvage pathway. The conversion of 2′-*O*-methyluridine into uracil could be performed by an unknown enzyme from the soil-based metagenomic libraries.

In order to search for genes supporting the growth of DH10BΔ*pyr* cells on M9 minimal medium supplemented with 2′-*O*-methyluridine (in this article abbreviated meUrd for clarity reasons, elsewhere abbreviated Um [1]), several metagenomic libraries were transformed into these cells. Subsequently, a single positive hit was selected and the pUC19 plasmid vector with a 2 kb insert was isolated and sequenced. This DNA fragment was named RK9 after the metagenomic library it was found in (GenBank accession number MK014213). The ensuing DNA sequence analysis using BLAST [BLAST: Basic Local Alignment Search Tool. Available online: blast.ncbi.nlm.nih.gov/Blast.cgi (accessed on 5 November 2018)] [31] revealed two ORFs that are facing in the same direction and are separated from each other only by 15 bp. The first ORF encodes an uncharacterized protein that is homologous to 2-deoxy-d-ribose 5-phosphate aldolase (RK9DPA), whereas the second ORF encodes an uncharacterized protein that is homologous to nucleoside hydrolases (RK9NH).

**Figure 2:**
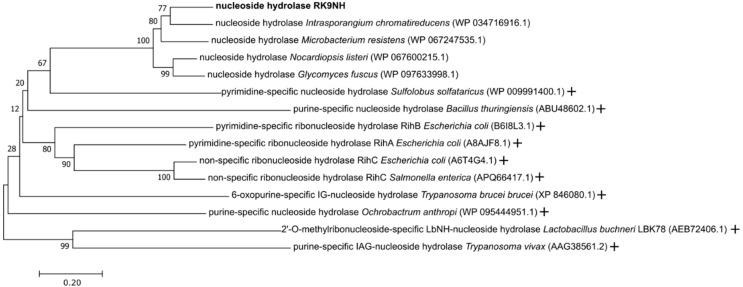
Evolutionary relationships of nucleoside hydrolases. The evolutionary history was inferred using the Neighbor-Joining method [36]. The optimal tree with the sum of branch length = 6.9 is shown. The percentage of replicate trees in which the associated taxa clustered together in the bootstrap test (1000 replicates) are shown next to the branches [37]. The tree is drawn to scale, with branch lengths in the same units as those of the evolutionary distances used to infer the phylogenetic tree. The evolutionary distances were computed using the Poisson correction method [38] and are in the units of the number of amino acid substitutions per site. The analysis involved 15 amino acid sequences. All positions containing gaps and missing data were eliminated. There were a total of 275 positions in the final dataset. Evolutionary analyses were conducted in MEGA7 [34]. RK9NH nucleoside hydrolase is shown in bold. The “+” indicates the enzymes with confirmed functions. The protein accession numbers are in the brackets.

The RK9NH amino acid sequence analysis [31] against the UniProt Knowledgebase [32] indicates that this protein is related to RihB, RihA and RihC hydrolases. RihA and RihB enzymes are cytidine/uridine specific hydrolases, while RihC is a non-specific purine/pyrimidine ribonucleoside hydrolase [33]. Thus, the phylogenetic analysis [34] of RK9NH nucleoside hydrolase (Figure 2) does not clearly place it among any well-known hydrolases. To no surprise, the phylogenetic analysis of RK9DPA deoxyribose-phosphate aldolase (Figure S1) does not clearly place it among any well-known aldolases as well. It is only distantly related to the 2-deoxy-d-ribose 5-phosphate aldolase (DERA) enzyme of *E. coli* [35] and is in a separate group from the aldolases with known structures.

**Figure 3:**
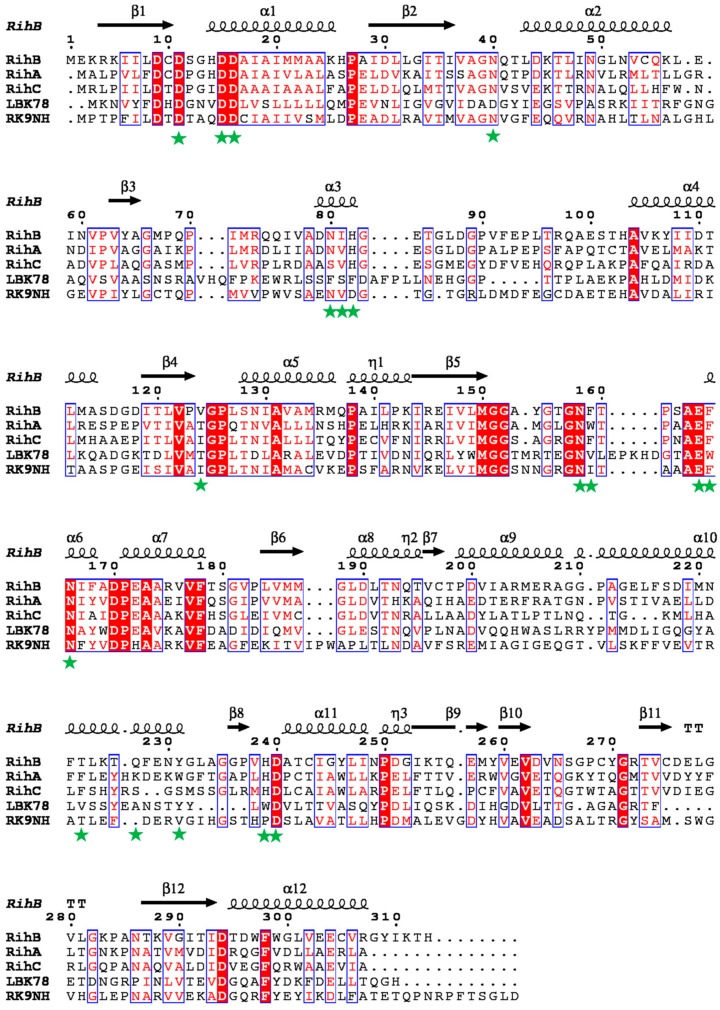
Multiple amino acid sequence alignment of nucleoside hydrolases. RihB: pyrimidine-specific ribonucleoside hydrolase from *E. coli* [39]; RihA: pyrimidine-specific ribonucleoside hydrolase from *E. coli* [33]; RihC: non-specific ribonucleoside hydrolase from *E. coli* [33]; LBK78: 2′-*O*-methylribonucleoside-specific nucleoside hydrolase from *Lactobacillus buchneri* LBK78 [27]; RK9NH: nucleoside hydrolase discovered in the metagenomic libraries. Highly similar residues are in red and framed in blue, strictly identical residues are in white on a red background. Green stars indicate the amino acid residues that form the active site of RihB [39]. The alignment was performed using Clustal Omega [40] and ESPript [41].

Multiple amino acid sequence alignment of RK9NH nucleoside hydrolase with RihB [33,39], RihA [33], RihC [33], and 2′-*O*-methylribonucleoside-specific nucleoside hydrolase from *Lactobacillus buchneri* LBK78 [27] (Figure 3) reveals that the amino acid residues that form the active site of RihB (Figure 3, green stars) are not all conservative in RK9NH, which suggests an even broader substrate specificity. It is also evident that RK9NH differs from *Lactobacillus buchneri* LBK78 nucleoside hydrolase, although these two enzymes share the same function.

### 2.2. Activity of RK9NH and RK9DPA Proteins in E. coli Cells

**Figure 4:**
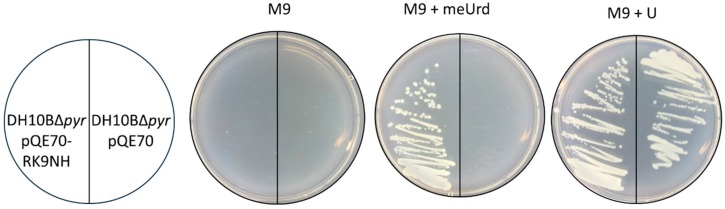
In vivo functionality of RK9NH coded in pQE70 vector. An empty vector was used as negative control. M9 was supplemented with 100 mg/L ampicillin, 15 mg/L kanamycin and either with 20 mg/L 2′-*O*-methyluridine (meUrd) or uracil (U, positive control).

Respective RK9DPA ORF and the RK9NH ORF were cloned into pQE70 expression vectors. As expected, it was determined (Figure 4, M9 + meUrd, left) that the RK9NH is responsible for the restoration of DH10BΔ*pyr* cell growth phenotype in minimal medium where the sole source of uracil is 2′-*O*-methyluridine. Nucleoside hydrolases cleave the *N*-glycosidic bond and, in this case, uracil is released.

The RK9DPA gene is related to deoxyribose-phosphate aldolases that catalyze a reversible aldol reaction between acetaldehyde and d-glyceraldehyde 3-phosphate to generate 2-deoxy-d-ribose 5-phosphate. It was tested whether RK9DPA has the same function, as does the deoxyribose-phosphate aldolase DERA from *E. coli*. A single-gene knockout mutant BW25113 *deoC*::*kan* strain (*deoC* gene encodes the DERA enzyme) from Keio collection [42] was transformed with pQE70-RK9DPA plasmid vector and grown in M9 minimal medium (Table 1). Thymidine at 2 mM concentration was used as the sole carbon source, because bacterial deoxyribose aldolases are known to be involved in the catabolism of deoxynucleosides arising from the dead cells, thereby giving an advantage to the microorganisms with a capability to consume DNA as an alternative carbon and energy source [43]. Usual glucose concentration was used as a carbon source for the positive control. Wild-type BW25113 strain was used as a positive control, BW25113 *deoC*::*kan* strain was used as a negative control.

The genetic complementation assay results suggest that RK9DPA is a different kind of an enzyme and does not have the same function as does the *E. coli* DERA enzyme.

### 2.3. Substrate Specificity of RK9NH Protein

The RK9NH protein has been cloned into pET21b(+) protein expression vector, overproduced in *E. coli* and purified (Figure S2). Substrate specificity assays were performed, and the results were analyzed using TLC and HPLC-MS.

**Figure 5:**
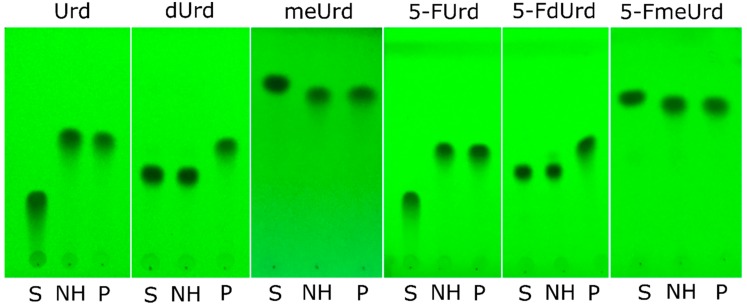
An example of Thin-Layer Chromatography (TLC) plates of substrates (S, also indicated above the plate), enzymatic RK9NH reactions (NH) and product standards (P): uracil is formed when uridine and 2′-*O*-methyluridine are used as substrates; 5-fluorouracil is formed, when 5-fluorouridine, 5-fluoro-2′-deoxyuridine and 5-fluoro-2′-*O*-methyluridine are used as substrates. S: substrate, NH: RK9 nucleoside hydrolase enzymatic reaction, P: product standards (uracil or 5-fluorouracil), Urd: uridine, dUrd: 2′-deoxyuridine, meUrd: 2′-*O*-methyluridine, 5-FUrd: 5-fluorouridine, 5-FdUrd: 5-fluoro-2′-deoxyuridine, 5-FmeUrd: 5-fluoro-2′-*O*-methyluridine.

**Figure 6:**
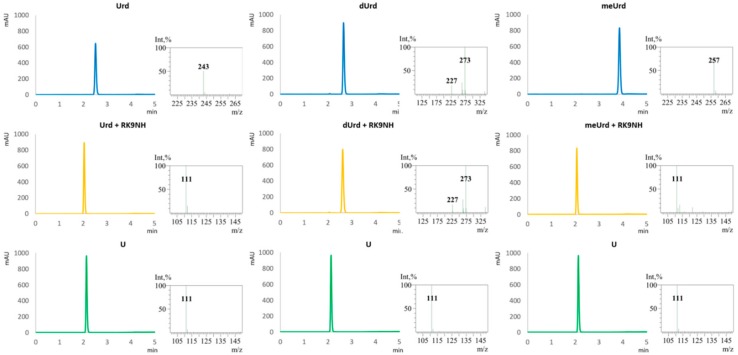
An example of the UV chromatograms (**left**) and MS-spectra corresponding to the peaks (**right**) of substrate standards (blue), nucleoside hydrolase RK9NH enzymatic reaction mixtures (yellow) and product standards (green). The *m*/*z* are consistent with [M-H]^−^ detection of molecular ions; the *m*/*z* 273 is consistent with [M + HCOOH-H]^−^ of 2′-deoxyuridine. Urd: uridine, dUrd: 2′-deoxyuridine, meUrd: 2′-*O*-methyluridine.

**Figure 7:**
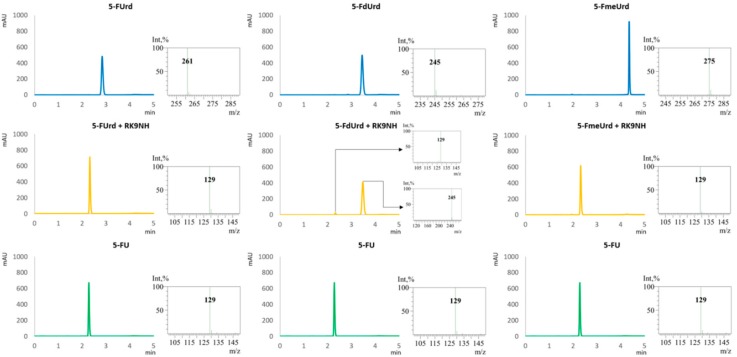
An example of the UV chromatograms (**left**) and MS-spectra corresponding to the peaks (**right**) of substrate standards (blue), nucleoside hydrolase RK9NH enzymatic reaction mixtures (yellow) and product standards (green). The MS spectra are consistent with [M-H]^−^ detection of molecular ions. 5-FUrd: 5-fluorouridine, 5-FdUrd: 5-fluoro-2′-deoxyuridine, 5-FmeUrd: 5-fluoro-2′-*O*-methyluridine, 5-FU: 5-fluorouracil.

The purified RK9NH has been tested for substrate specificity in vitro using a variety of different substrates, including the 5-fluoro derivatives of uridine and 2′-*O*-methyluridine hoping to detect 5-fluorouracil. After the reaction, the substrate, expected product and the reaction mixture were analyzed by TLC (Figure 5). Not all of the substrates and products are easily separated using TLC, therefore all of the reactions were analyzed with HPLC-MS. The HPLC-MS analysis results for the substrates seen on TLC plates in Figure 6 are provided in Figure 6 and Figure 7. The substrate specificity results with all of the substrates used are summarized in Table 2.

## 3. Discussion

It is a well-known fact that only very few microorganisms are cultivable in the laboratory. Thus, a large portion of genes and enzymes that exist in the environment are beyond the reach of scientists. The creation of metagenomic libraries (in this case the cloning of soil bacteria genomic DNA fragments into a plasmid vector that allows expression in *E. coli*) allows mining for novel enzymatic activities in a pool of these otherwise inaccessible genes. The soil metagenome was chosen, because this is a study of the basic metabolism of 2′-*O*-methyluridine, and the soil provides bacteria that are mostly not extremophiles. There is also a bigger chance that these bacterial enzymes will be expressed in *E. coli*, as the cell regulation might be similar. Also, the soil bacteria come into contact with metabolites of other life forms, and if the modified compounds are excreted from, e.g., animals, it is quite possible that soil bacteria can metabolize these readily-available compounds. This kind of function-based enzymatic screening of metagenomic libraries has been previously reported and reviewed [44].

Gene clustering is common in prokaryotic cells and helps to produce metabolic enzymes in a correct order. It is therefore quite possible that we discovered a part of a cluster of genes that encode enzymes involved in a metabolic pathway of 2′-*O*-methyluridine degradation of an unknown bacterium from a soil sample. This fragment of genomic DNA is similar to that of a Gram-positive, aerobic actinobacterium *Intrasporangium chromatireducens* (accession number NZ_AWQS01000091.1), except that the aldolase and hydrolase are separated by 908 base pairs in the genome of the aforementioned organism and a GntR family transcriptional regulator gene is in between the aldolase and nucleoside hydrolase genes. It was reported recently that a newly discovered bi-functional nucleoside hydrolase from *Agromyces* sp. MM-1 that catalyzes both hydrolysis of 2′-*O*-methylribonucleosides and transribosylation between 2′-*O*-methyluridine and various nucleobases, had 97% homology with a putative nucleoside hydrolase of *Microbacterium resistens*, which formed a gene cluster together with nucleoside-metabolizing enzymes such as 2-deoxyribose-5-phosphate aldolase, formamidopyrimidine-DNA glycosylase and ribokinase [29]. It is worth mentioning that a ribokinase gene is also present upstream of the aldolase coding gene in the genome of *Intrasporangium chromatireducens*.

According to the phylogenetic analysis (Figure 2) RK9NH is clearly neither a purine or pyrimidine specific hydrolase, nor it is placed near known non-specific hydrolases. It suggests that RK9NH is a unique hydrolase and that diversity exists among the nucleoside hydrolases involved in metabolism of 2′-*O*-methylribonucleosides. Most unexpectedly, RK9NH is not similar to a 2′-*O*-methylribonucleoside hydrolase from *Lactobacillus buchneri* (see Figure 3). This enzyme was discovered recently [27] and is already being used for the enzymatic synthesis of 2′-*O*-methylribonucleosides [28]. The phylogenetic analysis placed the RK9NH and the 2′-*O*-methylribonucleoside hydrolase from *L. buchneri* enzymes into separate groups (Figure 2).

It is clear from its substrate specificity (Table 2) that RK9NH acts essentially on the ribonucleosides as hydrolytic substrates with an exception of 5-fluoro-2′-deoxyuridine and 2′-deoxyguanosine, where the amounts of product formed are small, but detectable (see Table 2). Uridine, 2′-*O*-methyluridine, their 5-fluoro derivatives, 5-methyluridine, guanosine, cytidine and 2′-*O*-methylcytidine (to a lesser extent) were all accepted as substrates. The amount of the product formed when using inosine, adenosine, 2′-*O*-methyladenosine, and 2′-*O*-methylguanosine was small. There was no formation of uracil observed when 3′-*O*-methyluridine was used as a substrate for RK9NH. The 2′-*O*-allyl or 3′-*O*-allyluridine were also not accepted as substrates—suggesting that substrates with longer ribose side-chains are not suitable for the RK9NH enzyme. Substrate specificity suggests that RK9NH recognizes the 2′-hydroxy or the 2′-methoxy group in the sugar moiety of the substrate. The preferred nucleobases are uracil, 5-fluorouracil and guanine, but cytosine, inosine and adenosine were also acceptable. Based on these data, RK9NH falls between an unspecific and pyrimidine-specific nucleoside hydrolase that has a wide variety of substrates, with a preference to heterocyclic bases that are pyrimidine derivatives. Such modified nucleosides are of great interest lately, due to the possibility to use them for the synthesis of nucleic acid-based drugs. DNA or RNA oligomers with methylated nucleotides have high thermal stability and nuclease tolerance. Hence, nucleic acid drugs comprising modified DNA or RNA oligomers are the next-generation medicine with various applications such as antisense drugs, ribozymes, small interfering RNA drugs and aptamers [45]. Enzymatic synthesis of methylated nucleosides has only recently been achieved [28,29], which means that the RK9NH enzyme is the gateway to a new and promising field of biocatalysis. In addition, since 5-fluoro-2′-*O*-methyluridine is a good substrate of RK9NH hydrolase, a novel prodrug/enzyme combination might be considered as an addition to the existing systems generating 5-fluorouracil, a well-known anticancer compound [46,47,48].

## 4. Materials and Methods

### 4.1. Bacterial Strains, Plasmids, Primers, Media and Reagents

*E. coli* DH5α (Thermo Fisher Scientific, Vilnius, Lithuania) was used for routine DNA manipulations. *E. coli* DH10B (Thermo Fisher Scientific, Vilnius, Lithuania) was used for disruption of *pyr* genes. *E. coli* BL21(DE-3) (Novagen, Merck KGaA, Darmstadt, Germany) was used to produce the recombinant RK9NH nucleoside hydrolase protein. All of the DNA primers were synthesized at Metabion International AG, Munich, Germany. Standard techniques were used for DNA manipulations [49]. Vectors for inducible expression of C-terminally 6×His-tagged proteins pET21b(+) (Novagen, Merck KGaA, Darmstadt, Germany) and pQE70 (Qiagen, Hilden, Germany) were used for cloning of the RK9NH and RK9DPA genes. The RK9NH gene was amplified by PCR using primers *NH 21b FW* (5′-AAAACATATGCCGACCCCGTTCATC-3′) and *NH 21b RV* (5′-TTTTAAGCTTGTCGAGACCGGAAGTGAAC-3′), digested with *Nde*I and *Hind*III and cloned into the corresponding site of pET21b(+) vector, resulting in pET21b(+)-RK9NH plasmid vector. The RK9NH gene was amplified by PCR using primers *NH pQ Fw* (5′-AATAAGGATCCATGCCGACCCCGTTC-3′) and *NH pQ Rv* (5′-TATTAAGCTTTCAGTCGAGACCGGAAGTGAAC-3′), digested with *Bam*HI and *Hind*III and cloned into the corresponding site of pQE70 vector, resulting in pQE70-RK9NH plasmid vector. The RK9DPA gene was amplified by PCR using primers *DPA pQ Fw* (5′-TTAAGCATGCCTGACTTCCGATCGTTG-3′) and *DPA pQ Rv* (5′-GATGAGATCTGTAGCTCCGGGTGTCTTCG-3′), digested with *Pae*I and *Bgl*II and cloned into the corresponding site of pQE70 vector, resulting in pQE70-RK9DPA plasmid vector.

*E. coli* strains transformed with recombinant plasmids were grown in nutrient broth (NB) or Oxoid nutrient agar (NA) (Thermo Fisher Scientific, Vilnius, Lithuania) medium supplemented with either 100 mg/L ampicillin or 15 mg/L kanamycin, as required, at 37 °C with aeration (unless noted otherwise). *E. coli* DH10BΔ*pyr* cells transformed with metagenomic libraries were grown in M9 minimal medium with casamino acids [50] supplemented with 100 mg/L ampicillin, 15 mg/L kanamycin, 0.02 mg/mL uracil or 2′-*O*-methyluridine, as required, at 37 °C with aeration.

5-Fluoro-2′-*O*-methyluridine was synthesized and analyzed as described below. All of the remaining reagents, chemicals and kits used in this work are of the highest quality commercially available.

### 4.2. The E. coli Uracil Auxotroph Strain and the Metagenomic Libraries

The *E. coli* DH10BΔ*pyr* strain and the metagenomic libraries that were used in this study were described previously [30]. Briefly, the *pyrF*, *pyrE*, *pyrC* genes in DH10B strain of *E. coli* were disrupted using The Quick and Easy *E. coli* Gene Deletion Kit (Gene Bridges, Heidelberg, Germany) according to Version 2.3 of the technical protocol (June 2012).

The metagenomic libraries were prepared as described in [51] and used for transformation of *E. coli* DH10BΔ*pyr* electro-competent cells.

### 4.3. Synthesis of 5-fluoro-2′-O-methyluridine

A mixture of 200 mg (0.41 mmol) of commercially available 3′,5′-bis-*O*-benzoyl-5-fluoro-2′-*O*-methyluridine and 0.83 mL 1 M sodium methylate solution in methanol was stirred for 30 min at room temperature. The reaction was monitored with TLC (eluent methanol/chloroform; 1/9, *v*/*v*). After the reaction was completed, the mixture was neutralized with 1 M acetic acid. The crude reaction mixture was purified by reverse phase column chromatography (C-18 cartridges, water/methanol mixture, 10:0→10:2). The solvents were removed under reduced pressure to afford white solid reaction product. Yield 110 mg (97%). The purity of 5-fluoro-2′-*O*-methyl-uridine was analyzed by HPLC-MS and NMR spectroscopy methods, results of which are given bellow.

**5-Fluoro-2′-*O*-methyl-uridine**: MS (ESI^+^), *m*/*z* 275.05 (M − H)^−^, 277.05 (M + H)^+^. UV λ_max_ 269 nm. ^1^H NMR (DMSO-*d*_6_, 400 MHz): δ = 3.38 (s, 3H, CH_3_); 3.57–3.72 (m, 2H, CH_2_); 3.79 (t, 1H, *J* = 4.6 Hz, CH), 3.86 (m, 1H, CH); 4.13 (m, 1H, CH); 5.17 (bs, 1H, OH); 5.33 (bs, 1H, OH); 5.81 (dd, 1H, *J* = 4.3; 1.8 Hz, CH); 8.34 (d, 1 H, *J* = 7.3 Hz, CH=CF); 11.84 (bs, 1H, NH). ^13^C NMR (DMSO-*d*_6_, 100 MHz): δ = 58.03; 60.46; 68.40; 83.30; 85.39; 86.84; 125.21; 139.26; 149.41; 157.67.

### 4.4. Over-Expression and Purification of the Recombinant RK9NH Protein

RK9NH nucleoside hydrolase gene was separately cloned into pET21b(+) vector and transformed into the BL21(DE-3) cells. The resulting bacteria were grown in LB medium containing 100 mg/L ampicillin. The culture was grown at 37 °C until OD_600_ reached 0.5–0.6. It was then cooled on ice and the inducer isopropyl-1-thio-β-d-galactopyranoside (IPTG) was added. For recombinant RK9NH protein the final concentration of 1 mM IPTG was used and the induced cells were incubated at 37 °C for 3 h. The cells were then collected by centrifugation, resuspended in 50 mM TRIS-HCl, pH 8, and disrupted by sonication at 750 W for 1 min using a VC-750 ultrasound processor (Sonics & Materials, Inc., Newtown, CT, USA). Cell debris was removed by centrifugation at 16,000× *g* for 10 min. Cell extracts were loaded onto a Ni-NTA column (GE Healthcare Bio-Sciences, Upsala, Sweden) previously equilibrated with 50 mM TRIS-HCl, pH 8. The adsorbed proteins were eluted with 50 mM TRIS-HCl, pH 8 using linear gradient of 0–500 mM imidazole. The fractions containing the proteins were pooled and desalted by dialysis against 50 mM TRIS-HCl, pH 8. The purity of recombinant proteins was confirmed by electrophoresis on a 12% SDS-PAGE gel visualized by Coomassie Brilliant Blue (Thermo Fisher Scientific, Vilnius, Lithuania) staining (Figure S2). The concentration of recombinant proteins was measured using Lowry method [52] with bovine serum albumin as the standard.

### 4.5. Substrate Specificity Measurements

The purified recombinant RK9NH nucleoside hydrolase protein was tested for substrate specificity using the thin-layer chromatography (TLC) and high-performance liquid chromatography-mass spectrometry (HPLC-MS) methods. A standard enzymatic reaction of RK9NH was carried out at 37 °C for 1 h and contained 30 mM (uridine, 2′-deoxyuridine, 2′-*O*-methyluridine, 5-fluorouridine, 5-fluoro-2′-deoxyuridine, 5-fluoro-2′-*O*-methyluridine) of substrate, final 0.025 mg/mL concentration of recombinant 6×His-tagged RK9NH in 40 µL final volume of 50 mM Tris-HCl pH 8 buffer (the same results were obtained using a 50 mM potassium phosphate buffer pH 7). Not all of the substrates are well soluble in water (and the buffers used), hence some of the final reaction concentrations varied accordingly to the limit of solubility of the substrates: 20 mM cytidine, 2′-deoxycytidine, 5-methyluridine, thymidine; 15 mM 3′-*O*-methyluridine, 2′-*O*-allyluridine, 3′-*O*-allyluridine, 2′-*O*-methylcytidine, 2′-*O*-methyladenosine, 2′-*O*-methylguanosine; 10 mM adenosine, 2′-deoxyadenosine, 2′-amino-2′-deoxyuridine and 5 mM guanosine, 2′-deoxyguanosine, inosine were used. The final 0.017 mg/mL, 0.013 mg/mL, 0.008 mg/mL, 0.004 mg/mL RK9NH protein concentrations were used respectively.

### 4.6. Thin-Layer Chromatography (TLC)

1 μL of each enzymatic reaction sample was spotted onto an aluminum plate coated with silica gel 60 F_254_ (Merck Millipore, MA, USA) and developed with a solvent mixture as a mobile phase. Solvent system used for analysis of uracil, 5-fluorouracil, uridine, 2′-deoxyuridine, 5-fluorouridine, 5-fluoro-2′-deoxyuridine: methanol/chloroform (1/9, *v*/*v*). Solvent system used for analysis of 2′-*O*-methyluridine and 5-fluoro-2′-*O*-methyluridine: ethyl acetate/methanol/0.1 N hydrochloric acid (15/2/2, *v*/*v*/*v*) [28]. The spots were detected and visualized under the 254 nm UV light.

### 4.7. HPLC-MS

High-performance liquid chromatography–mass spectrometry (HPLC-MS) analyses were performed using a high-performance liquid chromatography system, equipped with a photo diode array detector (SPD-M20A) and a mass spectrometer (LCMS-2020), equipped with an electrospray ionization (ESI) source (Shimadzu, Kyoto, Japan). The chromatographic separation was conducted using a YMC Pack Pro column (YMC, Kyoto, Japan), 3 × 150 mm at 40 °C and a mobile phase that consisted of 0.1% formic acid water solution (solvent A) and acetonitrile (solvent B). Mass spectrometry data was acquired in both positive and negative ionization mode and analyzed using the LabSolutions LCMS software, version 5.42 SP6.

## Figures and Tables

**Table 1:** Complementation assay of *deoC*^−^ *E. coli* by transformation with pQE70-RK9DPA. Growth in single colonies was recorded as positive (+), no growth was recorded as negative (−), - < + < ++.

Strain	Carbon Source		
	None	Glucose	Thymidine
*E. coli* BW25113	-	++	+
*E. coli* BW25113 *deoC*::*kan*	-	++	-
*E. coli* BW25113 *deoC*::*kan* pQE70-RK9DPA	-	++	-

**Table 2:** Substrate specificity of hydrolysis by RK9NH. Conversions were defined as a percentage of the mass amounts of corresponding heterocyclic base (product) in the hydrolysis reaction mixture. The experiments were performed at least 3 times.

Substrate	Conversion (%)
Uridine	100
2′-Deoxyuridine	0
2′-*O*-methyluridine	100
3′-*O*-methyluridine	0
2′-*O*-allyluridine	0
3′-*O*-allyluridine	0
5-Fluorouridine	100
5-Fluoro-2′-deoxyuridine	4 ± 0.1
5-Fluoro-2′-*O*-methyluridine	100
2′-Amino-2′-deoxyuridine	0
Cytidine	65 ± 1
2′-Deoxycytidine	0
2′-*O*-methylcytidine	70 ± 1
5-Methyluridine	100
Thymidine	0
Adenosine	10 ± 0.2
2′-Deoxyadenosine	0
2′-*O*-methyladenosine	5 ± 0.1
Guanosine	100
2′-Deoxyguanosine	3 ± 0.1
2′-*O*-methylguanosine	5 ± 0.1
Inosine	22 ± 0.5

## Supplementary Materials

The following are available online at http://www.mdpi.com/1420-3049/23/11/2904/s1. Figure S1: Evolutionary relationships of aldolases, Figure S2: SDS-PAGE of recombinant 6×His-tagged RK9NH protein.
