# Supplementary material for: Identification of a 2′-O-Methyluridine Nucleoside Hydrolase Using the Metagenomic Libraries
Source: Molecules. 2018 Nov 7;23(11):2904. doi: 10.3390/molecules23112904 (PMC6278475; doi:10.3390/molecules23112904)
Supplement: Supplementary file 1 [file molecules-23-02904-s001.pdf]

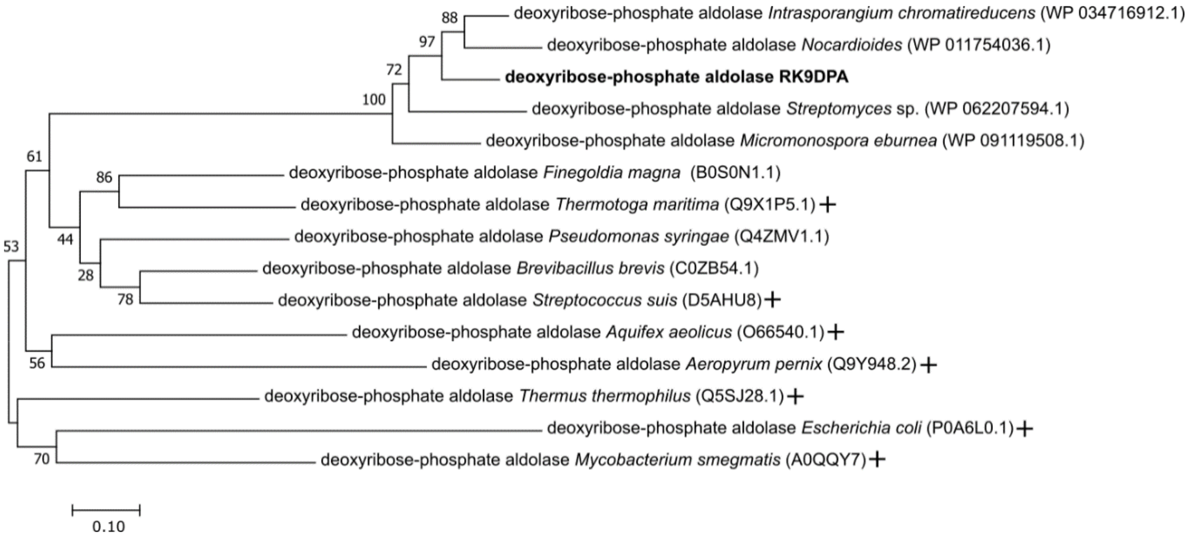

Figure S1. Evolutionary relationships of aldolases. The evolutionary history was inferred using the Neighbor-Joining method [32]. The optimal tree with the sum of branch length = 5.2 is shown. The percentage of replicate trees in which the associated taxa clustered together in the bootstrap test (1000 replicates) are shown next to the branches [33]. The tree is drawn to scale, with branch lengths in the same units as those of the evolutionary distances used to infer the phylogenetic tree. The evolutionary distances were computed using the Poisson correction method [34] and are in the units of the number of amino acid substitutions per site. The analysis involved 15 amino acid sequences. All positions containing gaps and missing data were eliminated. There were a total of 206 positions in the final dataset. Evolutionary analyses were conducted in MEGA7 [35]. RK9DPA aldolase is shown in bold. The "+" indicates the enzymes with confirmed functions. The protein accession numbers are in the brackets.

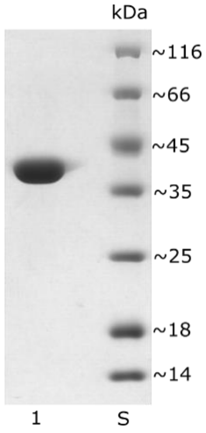

Figure S2. ~20 μg of recombinant 6xHis-tagged RK9NH protein (1) purified by Ni-affinity chromatography from cell-free extracts of *E. coli* BL21(DE-3) bacteria transformed with pET21b(+)-RK9NH plasmid that was induced with 1 mM IPTG 37°C for 3 h. (S) Pierce™ Unstained Protein Molecular Weight Marker (Thermo Scientific).
